# Supplementary material for: γ-Polyglutamic Acid Production, Biocontrol, and Stress Tolerance: Multifunction of Bacillus subtilis A-5 and the Complete Genome Analysis
Source: Int J Environ Res Public Health. 2022 Jun 22;19(13):7630. doi: 10.3390/ijerph19137630 (PMC9265942; doi:10.3390/ijerph19137630)
Supplement: Supplementary file 1 [file ijerph-19-07630-s001.zip › ijerph-1738397-supplementary.pdf]

Table S1. Analysis of the drug resistance genes in *B. subtilis* A-5.

Drug Class	Gene Number	Drug Class	Gene Number
macrolide antibiotic	33	isoniazid	5
fluoroquinolone antibiotic	33	triclosan	5
penam	26	lincosamide antibiotic	5
tetracycline antibiotic	19	nitroimidazole antibiotic	5
cephalosporin	19	glycopeptide antibiotic	4
peptide antibiotic	19	nucleoside antibiotic	3
cephamycin	15	streptogramin antibiotic	3
aminoglycoside antibiotic	12	nitrofuran antibiotic	2
carbapenem	11	pleuromutilin antibiotic	2
acridine dye	10	penem	2
monobactam	9	fosfomycin	2
aminocoumarin antibiotic	8	diaminopyrimidine antibiotic	1
rifamycin antibiotic	8	nybomycin	1
phenicol antibiotic	7	antibacterial free fatty acids	1
glycylcycline	5		

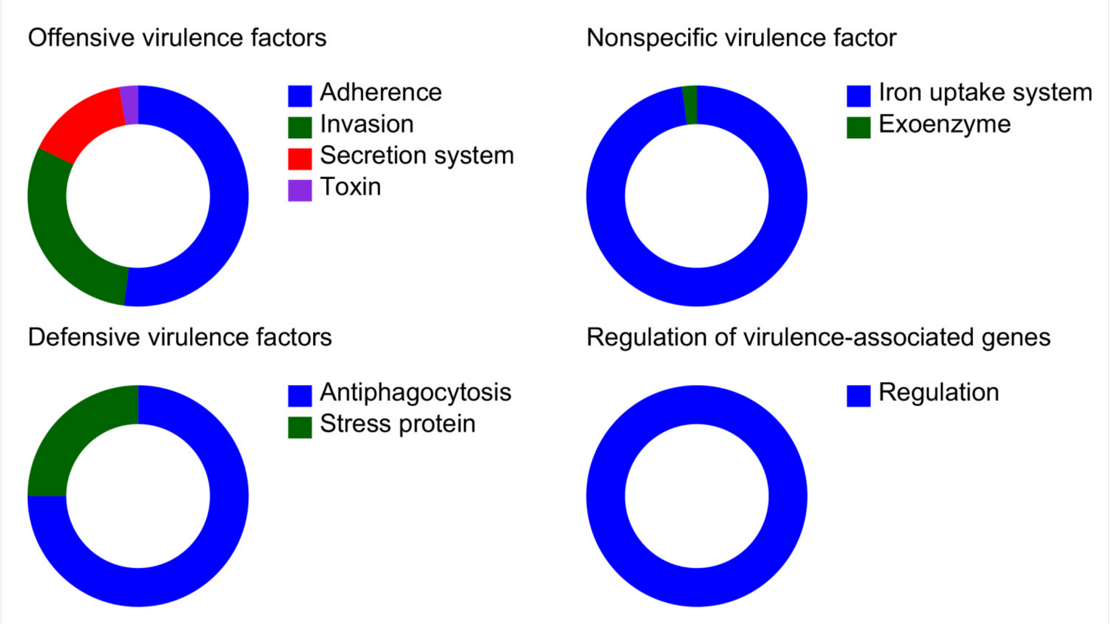

Figure S1. Analysis of the virulence factors in *B. subtilis* A-5 using VFDB database.

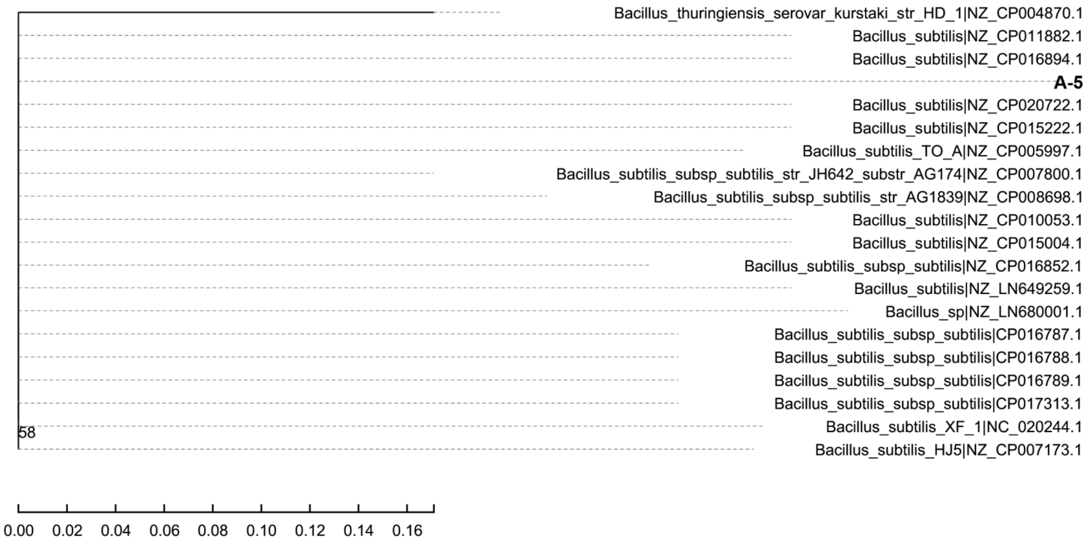

Figure S2 Phylogenetic tree of 30 house-keeping genes using the neighbor-joining method.
